# Dexmedetomidine as an Adjunct in a Fluoroscopically Guided Stellate Ganglion Block for Complex Regional Pain Syndrome

**DOI:** 10.7759/cureus.40504

**Published:** 2023-06-16

**Authors:** Haris Sheikh, Tanveer Baig

**Affiliations:** 1 Anaesthesiology, Aga Khan University Hospital, Karachi, PAK

**Keywords:** fluoroscopy, fluoroscopically-guided ganglion block, alpha 2 agonist, adjuvant to local anesthetic, dexmedetomidine, complex regional pain syndrome, stellate ganglion block (sgb)

## Abstract

Complex regional pain syndrome (CRPS) is a chronic pain disorder characterized by pain that is disproportionate to the inciting event. Autonomic and inflammatory responses predominate, and treatment plans that explicitly target these responses reduce symptoms for longer periods of time, are typically better tolerated, and have more favorable outcomes.

Our patient was a young male who presented with a four-month history of a road traffic accident (RTA) that resulted in a fractured left distal radius and scaphoid. His main complaint was pain and discomfort, even after surgical forearm stabilization, as well as hyperesthesia, restricted range of motion, and new-onset tremors. The patient was provisionally diagnosed with complex regional pain syndrome (CRPS) and booked for a fluoroscopically guided stellate ganglion block when the oral medication regime provided minimal relief. A stellate ganglion block was administered using a combination of ropivacaine, methylprednisolone, and dexmedetomidine under fluoroscopic guidance. During our routine outpatient follow-ups, our patient's pain score on the visual analog scale (VAS) fell to zero, the burning, vasomotor, and temperature abnormalities subsided, and he gradually regained the use of his left forearm and hand.

The etiology of complex regional pain syndrome is multifaceted. Early identification and therapy typically halt the progression. Long-term outcomes are improved by treatment strategies that target inflammatory and autonomic responses. Dexmedetomidine has a mild anti-nociceptive action when used as an adjuvant in peripheral nerve blocks and ganglion blocks, blocking pain transmission in Aδ and C fibers. We feel that by combining dexmedetomidine and a stellate ganglion block, we could provide immediate and long-term relief to our patient. More research is needed to monitor and analyze the efficacy of dexmedetomidine as a treatment for chronic pain syndromes such as CRPS.

## Introduction

Complex regional pain syndrome (CRPS) is a chronic pain condition characterized by spontaneous and evoked pain out of proportion to the inciting event [1]. Limited understanding of the mechanism and frequent lack of response to treatment have been major problems when dealing with patients with CRPS, which is why it is now being defined as a multifactorial process. However, the predominance of autonomic and inflammatory processes is often seen. Treatment strategies specifically targeting these responses alleviate symptoms for a longer period of time and are generally better tolerated with favorable outcomes [1].

Sympathetic blocks (stellate ganglion block and lumbar plexus block) have been beneficial in patients with CRPS [2]. Alpha-2 receptor agonists such as clonidine and dexmedetomidine have been clinically used for central and peripheral nerve blocks because of their local and central analgesic effects [3].

Here, we demonstrate the use of stellate ganglion block (SGB) in combination with dexmedetomidine to provide a patient with CRPS with long-term relief. 

## Case presentation

Our patient was a 28-year-old male with no known comorbidities who presented to the pain clinic with a history of a road traffic accident (RTA) four months ago, resulting in a left distal radius and left scaphoid fracture. He underwent open reduction and internal fixation (ORIF) of the left radius and scaphoid, along with an external fixator application. He also suffered a nasal bridge fracture, a minimal left temporal subdural hematoma, and a right arm and ear laceration, all of which were managed conservatively, and he was discharged in stable condition.

Following surgery, he reported moderate discomfort in the left forearm with a visual analog scale (VAS) score of six, which persisted throughout the day. The pain was searing in nature and occasionally felt like pins and needles, especially at night. As the ambient temperature dropped, his discomfort intensified. Additionally, he complained of reduced functionality of the left forearm and a lack of sleep due to pain.

On examination, he had redness and hyperesthesia in the left forearm. The range of motion of the left wrist and left forearm was decreased, and resting tremors were observed in the left hand that were not present before the RTA. A provisional diagnosis of CRPS was made, and the patient was counseled in detail about the nature of the disease and possible treatment options. The patient was given the following recommendations after an in-depth conversation and careful consideration: physiotherapy, neuromodulation with pregabalin 75 mg twice daily, duloxetine 30 mg once daily, and acetaminophen 1000 mg twice daily for 15 days. He was also advised to take etoricoxib 60 mg as needed for breakthrough pain. In addition, a fluoroscopically guided stellate ganglion block was scheduled for him, and if the pharmaceutical regimen did not relieve his symptoms, he was instructed to report for the block in 15 days.

**Figure 1:**
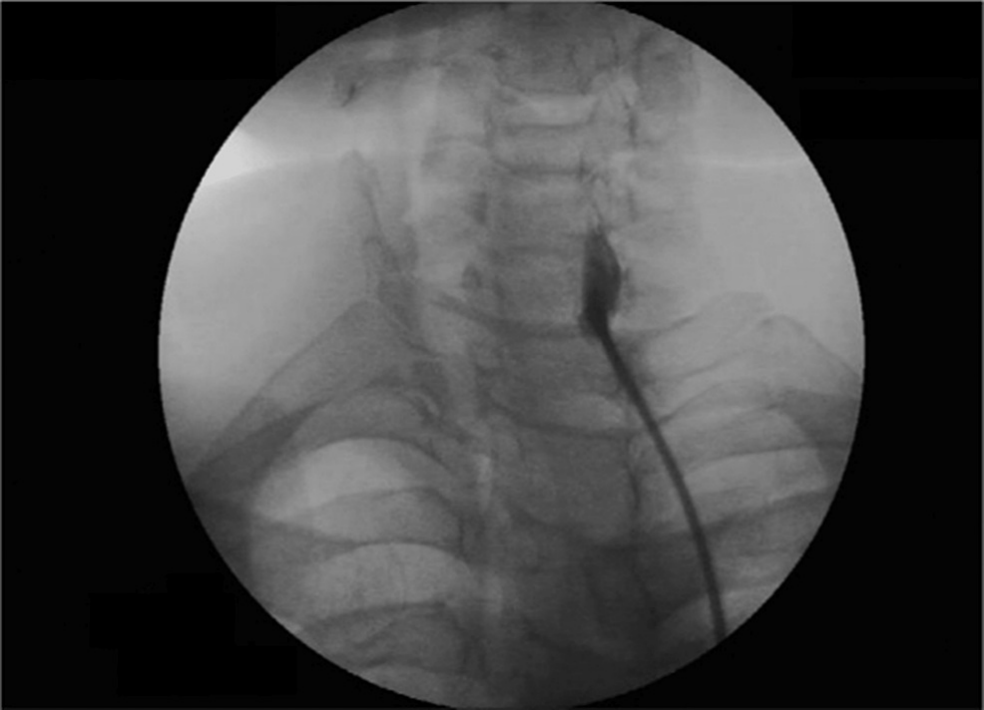
Fluoroscopic image of the dye spread at C6-C7 levels [4]

After 15 days, the patient sought to consult us for a stellate ganglion block because the medication provided minimal relief. A complete blood profile (CBC) and coagulation profile were ordered before the procedure. All aseptic measures were taken during the procedure in the operating room. An intravenous line was maintained before the procedure. Throughout the procedure, blood pressure, electrocardiogram (ECG), and pulse oximetry were monitored. Under real-time fluoroscopic guidance, the transverse process of the C7 vertebra was located, and the needle insertion site was numbed with 2% lidocaine, 2 ml. A 25-gauge, 44 mm long needle with a three-way stopcock was then inserted, and the needle position was verified by dye injection (Figure 1).

After negative aspiration, 4 ml of 0.5% ropivacaine, 40 mg of methylprednisolone, and 10 micrograms of dexmedetomidine were subsequently administered in a total volume of six milliliters. Throughout the procedure, the patient remained stable. After the procedure, he was transferred to the recovery room, where he experienced ipsilateral Horner's syndrome, which subsided in two hours. His VAS score decreased from six to two, and he was discharged from the hospital in stable condition.

The patient's VAS score fell to zero during our routine outpatient follow-ups. His burning and pins-and-needles-like sensations significantly decreased. Vasomotor and temperature changes abated, and his left forearm and hand gradually regained function. He has been receiving regular physiotherapy and has gone six months without experiencing any symptoms.

## Discussion

Complex regional pain syndrome arises because repeated painful stimuli create peripheral and central sensitization, an intensified inflammatory response, and autonomic nervous system involvement, which can lead to significant impairment and loss of function in the affected limb [1]. Patients with CRPS have clinically benefited from sympathetic blocks such as the stellate ganglion block and the lumbar plexus block on a large scale [2].

Because of their local and central analgesic characteristics, alpha-2 receptor agonists like clonidine have been employed as local anesthetic adjuncts for peripheral or central neuraxial blocks [3]. Dexmedetomidine is an alpha-2 agonist with eight times more affinity for alpha-2 receptors than clonidine. When used as an adjuvant in peripheral nerve and ganglion blocks, it has a modest anti-nociceptive action that blocks pain transmission in Aδ and C fibers and stops nerves from restoring resting potential from a hyperpolarized condition [5].

Brummett et al. combined dexmedetomidine and ropivacaine to block the sciatic nerve in rats, resulting in prolonged analgesia. The author hypothesized that the hyperpolarization-activated currents known as Ih (H current) regulate the hypothalamic paraventricular nucleus (PVN) neurons, which are near noradrenergic synapses. By preventing the Ih current from being activated, dexmedetomidine can keep cells in a hyperpolarized condition and improve the effects of local anesthetics. In unmyelinated C fibers (pain fibers) and small myelinated Aδ fibers (temperature sensitivity and fast pain sensation), this inhibitory impact is more pronounced [6].

Abd-Elshafy et al. used dexmedetomidine and bupivacaine for a paravertebral block during video-assisted thoracoscopic surgery (VATS) to provide superior acute pain management compared to bupivacaine alone. Three months following surgery, patients who received dexmedetomidine and bupivacaine together had a reduced incidence of chronic postoperative pain compared to those who only received bupivacaine [7]. After a modified radical mastectomy, Ali Hassn et al. employed dexmedetomidine and bupivacaine in a pectoral nerve block II and reported a significant decrease in both acute and chronic postsurgical pain at one, three, and six months [8].

Thapa et al. utilized dexmedetomidine as an adjuvant to lidocaine in the stellate ganglion block and found a significant decrease in tramadol use within 24 hours of surgery [9]. Recent research by Shrestha et al. suggests that ultrasound-guided SGB combined with dexmedetomidine can effectively treat CRPS [10].

Our patient was able to get significant and long-lasting pain relief by combining stellate ganglion block with dexmedetomidine. He was able to resume his regular activities after a notable improvement in his limb functionality. In addition, we were able to gradually reduce his oral medications to the point where he stopped taking them three months after the block. Alpha-2 receptor agonists may have the potential to treat CRPS or provide long-term relief with a single intervention by preventing sympatho-afferent coupling and reducing sympathetic outflow. This mechanism underlies the beneficial effects of the block with dexmedetomidine, which are caused by the activation of postsynaptic alpha-2 receptors, resulting in the inhibition of sympathetic activity [5]. It remains to be determined whether the various processes mentioned in this discussion assist in the management of chronic pain. Further studies are required to quantify and grade the effect of dexmedetomidine on chronic pain conditions such as CRPS [11].

## Conclusions

Complex regional pain syndrome is a complex illness with a wide range of potential treatments. As the etiology of CRPS is frequently related to autonomic responses, sympathetic blocks such as stellate ganglion blocks are among the most successful treatment modalities for the condition. However, local anesthetics alone are frequently found to be insufficient for lasting pain relief, and adjuvants such as dexmedetomidine, with favorable results in peripheral nerve blocks and ganglion blocks, may help alleviate pain and discomfort for a longer duration, as we found in our patient. We also used a steroid in our combination, which might have had a synergistic effect in breaking the pain cycle and providing long-term relief; however, steroids mainly help in reducing inflammation when used for local intervention procedures. Further studies are needed to determine the exact response of dexmedetomidine to treating chronic pain conditions such as CRPS.
